# Integrated Bioinformatic Analyses and Immune Characterization of New *Neisseria gonorrhoeae* Vaccine Antigens Expressed during Natural Mucosal Infection

**DOI:** 10.3390/vaccines7040153

**Published:** 2019-10-17

**Authors:** Tianmou Zhu, Ryan McClure, Odile B. Harrison, Caroline Genco, Paola Massari

**Affiliations:** 1 Department of Immunology, Tufts University School of Medicine, Boston, MA 02111, USA; tianmou.zhu@tufts.edu (T.Z.); caroline.genco@tufts.edu (C.G.); 2 Biological Sciences Division, Pacific Northwest National Laboratory, Richland, WA 99352, USA; ryan.mcclure@pnnl.gov; 3 Department of Zoology, University of Oxford, Oxford OX1 3SY, UK; odile.harrison@zoo.ox.ac.uk

**Keywords:** gonorrhea, vaccine, *in silico* analysis, hypothetical proteins, bactericidal antibodies

## Abstract

There is an increasingly severe trend of antibiotic-resistant *Neisseria gonorrhoeae* strains worldwide and new therapeutic strategies are needed against this sexually-transmitted pathogen. Despite the urgency, progress towards a gonococcal vaccine has been slowed by a scarcity of suitable antigens, lack of correlates of protection in humans and limited animal models of infection. *N. gonorrhoeae* gene expression levels in the natural human host does not reflect expression in vitro, further complicating in vitro-basedvaccine analysis platforms. We designed a novel candidate antigen selection strategy (CASS), based on a reverse vaccinology-like approach coupled with bioinformatics. We utilized the CASS to mine gonococcal proteins expressed during human mucosal infection, reported in our previous studies, and focused on a large pool of hypothetical proteins as an untapped source of potential new antigens. Via two discovery and analysis phases (DAP), we identified 36 targets predicted to be immunogenic, membrane-associated proteins conserved in *N. gonorrhoeae* and suitable for recombinant expression. Six initial candidates were produced and used to immunize mice. Characterization of the immune responses indicated cross-reactive antibodies and serum bactericidal activity against different *N. gonorrhoeae* strains. These results support the CASS as a tool for the discovery of new vaccine candidates.

## 1. Introduction

*Neisseria gonorrhoeae* is the causative agent of the sexually transmitted infection (STI) gonorrhea, a multi-faceted disease with high morbidity worldwide and an estimated 87 million cases annually [1]. *N. gonorrhoeae* infections in men are mostly symptomatic (urethritis), while gonorrhea in women is often asymptomatic, leading to reproductive tract complications (pelvic inflammatory disease (PID), ectopic pregnancy, infertility) and disseminated gonococcal infections (DGI) [2]. Once easily treated by a standard antibiotic course, current therapeutic and pharmacologic approaches for *N. gonorrhoeae* infections are now complicated by the onset of fluoroquinolone resistance and rising levels of resistance to the last FDA-approved antibiotic, cefixime [3,4,5]. Presently, the CDC recommends treatment with ceftriaxone and azithromycin, but resistance to cefixime and ceftriaxone has already developed outside the U.S., leading to the potential spread of untreatable gonorrhea [1]. Repeated gonococcal exposure may result in some strain-specific immunity but protective memory responses are scarce [6,7,8]. There is an urgent need to develop a vaccine to prevent gonococcal infections. 

Killed whole organisms and purified gonococcal pilin have been tested in vaccine clinical trials in human volunteer male urethral infections [9,10,11], a model limited to assessing acute infection that does not replicate chronic female reproductive tract infections [12]. Both vaccines failed to induce protection against heterologous re-infection, explained by antigenic and phase variability of pilin [13,14], and by induction of a robust antibody production to Rmp (reduction modifiable protein, a highly conserved major gonococcal surface antigen), which inhibited complement-mediated bacterial killing activity of antibodies elicited to other antigens in the whole gonococcal vaccine (for example, porin) [15,16,17]. Some protection was shown by antibodies to the gonococcal lipoligosaccharides (LOS) and a porin-based vaccine was also explored [2] but, like pilin, porin is subject to immunological pressure [18,19,20] and also affects complement-dependent bacterial killing [21]. Pre-clinical vaccine studies have been carried out in mouse models of immunization and infection [22,23] using classical surface-exposed antigens (for example, the highly conserved LOS epitope 2C7 [24], transferrin binding proteins (TbpA and TbpB) [25,26]), conserved and/or variable proteins (reviewed in Reference [2]) identified by conventional screenings, reverse vaccinology (initially developed for the *Neisseria meningitidis* serogroup B vaccine 4CMenB [27,28]), “omics” and bioinformatics [29,30,31,32,33,34,35]. Interest in outer membrane vesicles (OMVs) has been recently renewed by evidence of cross-reactive protection against *N. gonorrhoeae* by a meningococcal OMV-based vaccine [36,37,38,39]. However, evaluation of gonococcal vaccines is complicated by the lack of known correlates of protection against natural mucosal infections in humans [2]. Mechanisms of protection may include antibody-dependent complement-mediated killing (i.e., bactericidal activity and opsonophagocytic activity), inhibition of adhesion/invasion at the site of colonization and T cell responses, but none of these have been confirmed in human studies [40]. The antibody bactericidal activity assay (SBA) is often used as a surrogate of protection for gonorrhea in pre-clinical vaccine studies, mostly based on the experience with meningococcal infections [41,42].

We recently reported the gonococcal transcriptome expressed during natural human mucosal infection in men and women [43,44]. Our studies highlighted three important observations: (1) the gonococcus specifically responds to the male and female reproductive tract environments by expressing genes at different levels, (2) gonococcal genes are expressed and regulated differently in vivo and in vitro (genes detected as more expressed in vivo than in vitro were termed IVEFs (in vivo expressed factors)) and (3) a large number of gonococcal genes expressed during human infection encode hypothetical proteins. Taking this information into account, we have designed a comprehensive, high-throughput *in silico* approach for vaccine antigen identification that combines gene expression levels with known antigen prerequisites such as immunogenicity, membrane association/surface exposure, conservation and structure features. This candidate antigen selection strategy (CASS) is applicable to any protein group; here we focused on the gonococcal hypothetical proteins as an untapped pool of potential new antigens. By implementing two discovery and analysis phases (DAP), we obtained a subset of 36 targets predicted to be potential new antigens. Validation of the predicted features and immune-characterization of six initial candidates is presented. Our results show induction of cross-reactive antibodies against diverse *N. gonorrhoeae* strains by the candidates and serum bactericidal activity. These initial studies support the CASS for identification of gonococcal proteins not previously explored as vaccine antigens.

## 2. Materials and Methods 

### 2.1. Candidate Antigen Selection Strategy (CASS) and Computational Tools

Bioinformatic analyses and prediction tools were used with default parameters to analyze and prioritize the candidate antigens: VaxiJen, for antigenic and protective potential prediction [45] (cut-off of 0.4, as previously described [29]); PSORTb v3.0, PredictProtein and Gneg-mPLoc, for protein subcellular localization [46,47,48]; Vaxign [49] and BLASTp [50] for protein sequence analyses (similarity with human, mouse, *Escherichia coli*, *N. gonorrhoeae* and *lactamica*) [51,52]; TMHMM v.2.0, for presence and number of trans-membrane domains [53]; Phobius, for protein topology [54]; SignalP v5.0 and SecretomeP v2.0, for presence/type of signal peptides, cleavage site and post-translational modifications [55,56,57]. Protein functions were cross-checked with BLAST, UniProtKB, PFAM v32.0 and KEGG v90.1 [58,59,60] and PubMed records in NCBI. Protein sequence conservation with *N. gonorrhoeae* FA1090 (Gen Bank accession number AE004969) and within the available *N. gonorrhoeae* protein database was examined by BLASTp. Gene presence, alleles distribution and sequence conservation were examined in *N. gonorrhoeae* (4198 strains) and *N. lactamica* (288 strains) in PubMLST [61,62]. 

### 2.2. Bacterial Strains and Growth Conditions 

*N. gonorrhoeae* strains F62 and FA1090 were used as laboratory reference strains and two *N. gonorrhoeae* strains, U08401 and U08402, representative strains isolated from an infected male and his self-reporting monogamous female partner, respectively, in our Nanjing cohort strain collection [43,44]. Bacteria were grown overnight on GC agar plates supplemented with 1% IsoVitaleX at 37 °C with 5% CO_2_ or in liquid culture in chemically defined medium (CDM) for 1–3 h (O.D.)_600_ of 1 = 1–2 × 10^9^ bacteria/mL) and resuspended at the desired concentration for each experiment. For formalin-fixing (FF), bacteria were incubated with 1% paraformaldehyde for 1 h at 4 °C, washed and resuspended in PBS. *E. coli* strain BL21 was grown on LB agar plates at 37 °C with 5% CO_2_ or in LB liquid cultures using kanamycin (50 µg/mL) or carbenicillin (100 µg/mL) as selection antibiotics. 

### 2.3. Cloning and Expression of Recombinant Gonococcal Hypothetical Proteins 

Cloning was outsourced to GenScript (Piscataway, NJ, USA). Primers were designed based on the available protein sequences of *N. gonorrhoeae* FA1090 in NCBI: NGO0416 (YP_207571.1), NGO0690 (YP_207829.1), NGO0948 (YP_208051.1), NGO1043 (YP_208127.1), NGO1215 (YP_208286.1) and NGO1701 (YP_208734.1). All proteins except for NGO0948 were cloned in a pET17b plasmid as truncated proteins without the signal sequence with an ampicillin-resistance cassette using NdeI and HindIII restriction sites and included a C-terminal 6x His-tag. NGO0948 was cloned in a pET30a plasmid as a full-length protein with kanamycin resistance cassette and included a N-terminal 6x His-tag. The constructs were transformed into *E. coli* BL21 (DE3) for recombinant expression, NGO0416 and NGO1043 were expressed by growing cultures in LB broth with the appropriate antibiotics and 1 mM IPTG overnight at 22 °C, and NGO0690, NGO0948, NGO1215 and NGO1701 were expressed by 1 mM IPTG induction for 4 h after overnight growth at 37 °C. 

### 2.4. Chromatography Purification of Recombinant Hypothetical Proteins

Bacterial suspensions were centrifuged (3800× *g* for 15 min at 4 °C), the pellets were resuspended in 3 mL of Buffer A (50 mM sodium phosphate, 300 mM NaCl, pH 7.8, protease inhibitor cocktail (P8340, Sigma, St. Louis, MO, USA)) per gram of cells, and lysed with lysozyme and deoxycholate (0.1 mg/mL and 4 mg/g of cell pellet) at room temperature. DNAse I (0.02 mg/g of cell) was added until the suspension became non-viscous, and the soluble protein fraction and the inclusion bodies were separated by centrifugation (12,000× *g* for 20 min at 4 °C). All proteins were purified on a Ni^++^ fast-flow agarose resin column using an AKTAprime plus chromatography system (GE). NGO1043, NGO1215 and NGO1701 were purified from the soluble fraction in Buffer A with a 10–300 mM imidazole gradient. NGO0416, NGO0690 and NGO0948 inclusion bodies were solubilized in Buffer A with 10 mM imidazole and 6 M guanidine and purified in Buffer B (50 mM sodium phosphate, 300 mM NaCl, pH 7.8, 1× protease inhibitor cocktail (Sigma, #P8340)) with 10 mM imidazole and 8 M urea and a 10–300 mM imidazole gradient. The column flow-through, wash and elution fractions were examined by SDS-PAGE/Coomassie staining to assess protein purity and by dot blot using a mouse anti-His tag horse radish peroxidase (HRP)-conjugated antibody (Invitrogen, Carlsbad, CA, USA) and 1-Step TMB (3,3′,5,5′-tetramethylbenzidine) detection (Thermo Fisher Scientific) to identify the positive fractions. These were pooled and extensively dialyzed against PBS/0.02% NaN_3_. The protein concentration was measured by BCA (bicinchoninic acid) assay (Thermo Fisher Scientific). 

### 2.5. Immunization of Mice 

Female BALB/c mice (4–6 weeks old) (Jackson Labs, Bar Harbor, ME, USA) were housed and cared for according to NIH and Tufts University protocols under standard temperature and humidity, a 12-h lighting cycle, food and water available *ad libitum*. Experiments were carried out under protocols approved by the Tufts ethics committee (IACUC protocol B2018-88) and all efforts were taken to minimize pain and discomfort to the animals. Groups of eight mice were immunized subcutaneously (s.c.) three times at two-week intervals with purified recombinant proteins (10 µg/mouse/immunization) and alum (Imject) (Thermo Fisher Scientific) at a 1:1 v/v ratio in a total volume of 100 µL/immunization. A control group was immunized with PBS and alum (adjuvant control). Pre-immune sera were collected prior to the first immunization and immune sera were collected two weeks after each immunization. Sera were stored at −80 °C until use.

### 2.6. Immunoblotting 

Formalin-fixed (FF) bacteria (2–4 × 10^8^ CFU total) or outer membrane protein (OMPs) fractions (5 µg total) isolated using Sarkosyl as previously described [34] were spotted on a nitrocellulose filter using a slot-blot apparatus (Thermo Fisher Scientific). The membranes were blocked with 1% BSAin TBS/Tween 20 (TBS-T) and incubated with pooled sera aliquots (1:200 dilution) overnight at 4 °C. An anti-mouse IgG secondary AP-conjugated antibody (Southern Biotech, Birmingham, AL, USA) was used to detect immunoreactive dots with NBT/BCIP (5-bromo-4-chloro-3-indolyl phosphate/Nitroblue Tetrazolium) chromogenic substrate (Bio-Rad, Hercules, CA, USA). 

### 2.7. Antibody ELISA 

ELISA plates were coated with 2 μg/well of purified proteins in carbonate buffer pH 9.0 or with FF *N. gonorrhoeae* (1–1.5 × 10^7^ bacteria/well) in PBS overnight at 4 °C. Plates were washed, blocked with 1% BSA in PBS/0.05% Tween-20 for 2 h at room temperature and incubated overnight at 4 °C with serial dilutions of pre-immune and immune sera. The next day, plates were washed, incubated with AP-conjugated secondary anti-mouse total IgG, IgG1 or IgG2a antibodies (Southern Biotech) followed by 1-step PNPP (p-nitrophenyl phosphate) reagent (Thermo Fisher Scientific) and spectrophotometric detection at O.D._405_. Sera from individual mice were tested in duplicate wells, and pooled sera aliquots were tested in a minimum of triplicate wells. IgG and IgG subclasses were quantified in µg/mL using antibody reference standard curves (Southern Biotech, #5300-01) and a linear regression function [63]. The Th2/Th1 ratio was determined as IgG1 (µg/mL)/IgG2a (µg/mL). 

### 2.8. Cytokine ELISA 

IL-4 and IL-10, IL-12 and IFN-γ, IL-6 and TNF-α were measured in pooled sera aliquots by ELISA using Opt-EIA kits (BD Biosciences, San Jose, CA, USA) according to the manufacturer’s specifications. Cytokines were expressed in pg/mL.

### 2.9. Flow Cytometry 

FF *N. gonorrhoeae* (10^8^/mL) were incubated with pooled sera aliquots (1:200) in 2% FBS/PBS for 30 min at 4 °C, washed and stained with an anti-mouse IgG fluorescein isothiocyanate( FITC)-labeled secondary antibody (eBioscience) (1:1000) for 30 min at 4 °C. Controls included bacteria alone or incubated with only FITC-labeled secondary antibody. Samples were examined on a FACScan^(TM)^ flow cytometer using CellQuest acquisition software (Becton Dickinson, Mountain View, CA, USA) and analyzed with FlowJo software (Tufts University Flow Cytometry Core). Gating was used to exclude cellular debris. Histograms are representative of a minimum of two separate experiments. 

### 2.10. Bactericidal Assay (SBA) 

The SBA was carried out in 96-well U-bottom plates in a 75 µL total volume. Normal human serum (NHS) depleted of IgG and IgM was used as a source of complement (Pel-Freez Biologicals, Rogers, AR, USA). *N. gonorrhoeae* liquid cultures at OD_600_ of 0.2 (2–4 × 10^8^ CFU/mL) were serially diluted to 2–4 × 10^4^ CFU/mL and 12.5 µL of suspension/well were placed in HBSS containing 0.15 mM CaCl_2_ and 1 mM MgCl_2_ (HBSS^++^). The bacteria were then incubated with serial dilutions of heat-inactivated (56 °C for 30 min) pre-immune or immune pooled sera aliquots (previously depleted of the IgM fraction) for 20 min at room temperature. Then NHS was added to each well (20% for FA1090, 10% for F62, U08401 and U08402) and 10 µL of the bacterial suspension were immediately plated in duplicate on IsoVitalex-GC agar plates (Time 0). The 96-well plates were incubated at 37 °C for 30 min with gentle shaking, and aliquots were plated as above (Time 30). The next day, bacterial killing was evaluated by CFU counting; survival was expressed as percent of CFUs at T30/T0 ± SEM. The bactericidal titer was defined as the reciprocal of the lowest serum dilution with ≥50% killing after 30 min [64]. Controls included bacteria alone and bacteria incubated with NHS alone. 

### 2.11. Statistical Analysis 

Statistical significance was examined with GraphPad Prism 7.04. For comparison between two samples or conditions, an unpaired t-test was used to determine significance, and for comparisons between multiple groups, one-way analyses of variance (ANOVA) with Tukey’s multiple comparisons or with Dunnett’s tests were used. Differences were considered significant at a minimum *p* value of 0.05, as indicated in the text and in the Figure legends.

## 3. Results

### 3.1. Gonococcal Candidate Antigen Selection Strategy (CASS) 

**Figure 1:**
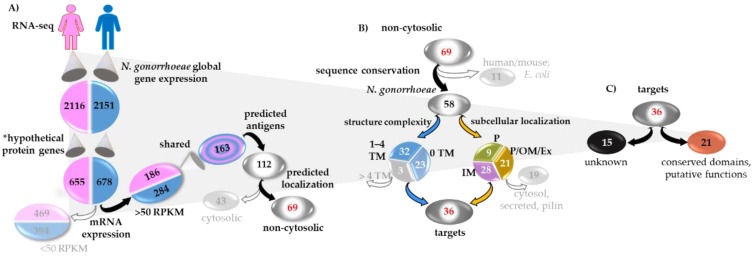
Candidate antigen selection strategy (CASS). (**A**) Discovery and analysis phase 1 (DAP-1): gonococcal hypothetical proteins (*Rockhopper analysis/NCBI annotation) expressed during natural human mucosal infections included 163 proteins with mRNA expression above 50 reads per kilobase of transcript per million mapped reads (RPKM) shared in infected male and female specimen datasets. *In silico* analysis predictions: antigenic potential, 112 proteins; cellular localization, 43 cytosolic proteins (grayed out) and 69 non-cytosolic proteins. (**B**) DAP-2, *in silico* analysis predictions: amino acid sequence conservation, 11 proteins conserved in human, mouse and *E. coli* (grayed out), 58 proteins conserved in *N. gonorrhoeae*; structure predictions: three proteins with >4 trans-membrane domains (TM) (grayed out), 32 with 1–4 TMs and 23 with no TMs; sub-cellular localization: 28 inner membrane (IM) associated proteins, nine periplasmic (P), 21 outer membrane (OM) (facing the periplasm or the extracellular space, P/OM/Ex) proteins; topology/signal peptide: IM, nine cytosolic topology/function, one secreted, four pilin-associated proteins; OM/P/Ex, two secreted, three pilin-associated proteins (grayed out). (**C**) CASS target annotation: 15 uncharacterized proteins, 21 proteins with conserved domain similarity to proteins with a defined function, a putative function or a newly-described function.

A multi-pronged candidate antigen selection strategy (CASS) was designed for antigen mining by screening predicted immunogenicity, surface-exposure, sequence conservation/variability and structure features combined with expression levels. The CASS was applied to gonococcal proteins classified as hypothetical in our previous gonococcal transcriptome study [43]. Briefly, gonococcal gene expression in urethral and cervico-vaginal lavage specimens from a cohort of naturally-infected subjects attending the National Center for STD Control (NCSTD) in Nanjing, China [43,44], was determined by RNAseq and reported in RPKM (RNA-seq gene expression = unit of transcript expression in RPKM or Reads per Kilobase of transcript per Million mapped reads). Within the total gonococcal genes expressed in these datasets, approximately 30% encoded for proteins annotated as hypothetical or putative by Rockhopper based on the NCBI database [43] (Figure 1A). 

#### Discovery and Analysis Phase (DAP)

In DAP-1, the hypothetical proteins were segregated in two groups based on gene expression levels. Proteins with RPKM >50 (186 in the female dataset and 284 in the male dataset) were selected and matched to identify those shared in both datasets, corresponding to 163 proteins (Figure 1A). Proteins with RPMK <50 (469 in the female dataset and 394 in the male dataset) were excluded at this time (Figure 1A, grayed out). Next, high throughput bioinformatic tools were applied; first, analysis with VaxiJen [45] (cut-off threshold of 0.4 [29]) to determine antigenic probability. This tool assigns an unbiased antigen probability score based on a protein’s amino acid sequence and predicted 112 putative antigens (Figure 1A). PSORTb (v3.0), PredictProtein and Gneg-mPLoc [46,47,48] predicted 43 proteins to be cytosolic, which were excluded at this time, and 69 non-cytosolic proteins that were advanced to DAP-2 (Figure 1B). Analysis of amino acid sequence conservation with human, mouse and *E. coli* proteins with Vaxign [49] and BLASTp [50,51,52] predicted three proteins with E-value >1 × 10^−10^ versus human/mouse counterparts, and eight proteins with >40% sequence similarity over >70% of the sequence length in *E. coli* proteins. These 11 proteins represented potential autoantigens and widespread bacterial housekeeping genes and were excluded. The remaining 58 proteins had a predicted >40% sequence similarity over >70% of the sequence length within the available *N. gonorrhoeae* protein databases in NCBI (Figure 1B). Next, TMHMM v.2.0 analysis [53] predicted 32 proteins with no trans-membrane domains (TM), 23 proteins with 1–4 TMs (considered low/moderate structure constraints) and three proteins with >4 TM domains, which were excluded due to potential difficulty of recombinant expression and purification (Figure 1B). The predicted membrane localization was parsed in greater detail, aided by topology and signal peptide predictions with Phobius, SignalP v5.0 and SecretomeP v2.0 [54,55,56,57]: 21 proteins were predicted to be associated with the outer membrane, either facing the periplasm or the extracellular space (P/OM/EX), nine were likely periplasmic (P) and 28 were predicted to be associated with the inner membrane (IM) (Figure 1B). The latter were further revised by PSI-Blast and manually curated to exclude those possibly involved in cytosolic processes (nine proteins). Protein post-translational structure modifications were also considered, and predictions of presence and type of signal peptide (SP) indicated that 20 proteins did not possess a SP sequence, 16 contained a classical SP sequence, seven contained a TatP-predicted SP, five were non-classically exported membrane proteins (special folding requirements or part of large protein complexes) and 10 proteins were predicted outer membrane-exposed lipoproteins (although this was based on the +2 amino acid position after the cleavage site established in *E. coli* [65]). Thus, three likely extracellular proteins and seven putative pilin-associated proteins were excluded from the IM and OM proteins, resulting in a final pool of 36 candidates (Figure 1B). Further interrogation within the BLAST, UniProtKB, PFAM and KEGG databases, potential functions were attributed to 21 targets by either conserved domain (CD) similarity to other bacterial proteins with defined function, putative functions or newly described functions, while 15 proteins remained fully uncharacterized (Figure 1C).

To begin exploring these targets, we started from six proteins based on a range of expression levels (higher in female specimens, higher in male specimens or equivalent levels), predicted antigenicity, structure complexity and potential evidence of function. The candidates were: (1) NGO0416, a hypothetical P protein with CD similarity to the N-terminal domain of the LamB sugarporin [66]; (2) NGO0690, a putative P/OM lipoprotein, possibly involved in threonine biosynthesis and pilin antigenicity [67]; (3) NGO0948, a P/OM lipoprotein member of the NlpB/DapX family (COG3317), homolog to BamC (a member of the BAM complex [68], surface-exposed in *E. coli* [69]) and a potential meningococcal vaccine antigen (NMB0928 [70,71,72,73]); (4) NGO1043, a hypothetical P/OM putative lipoprotein, possibly glycosylated and a substrate for phosphoethanolamine (PE) addition [74,75], with homology to the meningococcal antigen Ag473 [76]; (5) NGO1215, a hypothetical P protein with homology to a copper chaperone PCu(A)C superfamily (COG2847) (potential virulence factors) [77] and reported as AccA [78], and (6) NGO1701, a P membrane protein with homology to a TAT_Cys_rich four helix bundle copper-binding protein of the DUF326 superfamily. Table 1 summarizes the major CASS features for these candidates. 

### 3.2. Sequence Analysis and Conservation 

The six candidates were examined for gene sequence presence and conservation in a total of 4198 gonococcal genomes available in the PubMLST database [61,62] This was a globally representative collection of *N. gonorrhoeae* strains spanning six decades (1960–2018) and included strains from the Nanjing collection. The candidates were present in all gonococci examined and were part of the gonococcal core genome (Ng cgMLST v1.0). The corresponding NEIS nomenclature for these proteins is shown in Table 2. Differing numbers of alleles were identified, indicating some sequence diversity: the most conserved gene was *ngo0416* with only six alleles (*p*-distance = 0.006), while the most diverse was *ngo0948* (*p*-distance = 0.005) with 55 alleles, although alleles could not be assigned for eight strains, due to incomplete sequence data as genes were located at the end of contigs. This was also the case for *ngo1215* in two isolates, and 27 strains had incomplete *ngo1043* genes, a 21bp repeat region at nt 127 (5’- GCCGCCGAGTCTGCGGCTTCT-3’) was present three times in allele 138, twice in allele 137 and once in the remaining *ngo1043* alleles except allele 141. Due to the large number of alleles for some genes, only those corresponding to the most represented ones (above 5% in the total strains) are shown in Table 2. For example, *ngo0416* allele 17 was present in 3069 *N. gonorrhoeae* strains out of the 4198 total strains, corresponding to 73%; *ngo0416* also contained five polymorphic sites with two non-synonymous amino acid sequence mutations but these were not found in most represented alleles (Table 2). *ngo0690* possessed 27 polymorphic sites with 16 total nonsynonymous residues, one of which was in the high-frequency allele 32 (Table 2); *ngo0948* has 109 polymorphic sites with 54 nonsynonymous residues, *ngo1043* had 26 polymorphic sites with 13 nonsynonymous residues, 27 polymorphic sites with 14 nonsynonymous residues were identified in *ngo1215* and 10 with eight nonsynonymous residues in *ngo1701* (Table 2).

An expanded BLASTp amino acid sequence analysis against the human commensal *N. lactamica* protein database in NCBI revealed that 18 of the 36 candidates had <60% amino acid sequence similarity over the entire protein length, including NGO0416 and NGO1215. Gene presence and distribution of the six candidates in the *N. lactamica* genomes (a total of 288 genomes) in the PubMLST database was examined, revealing that *ngo0416* was absent in *N. lactamica* and no allelic overlap with gonococci for the other five hypothetical protein genes (Table S1). Comparison of the most prevalent alleles from each species for each locus revealed low conservation. 

### 3.3. Network Analysis

We performed a complementary analysis of gene expression in correlation with growth conditions by merging our transcriptome data with a collection of additional RNA-seq datasets derived from different and multiple *N. gonorrhoeae* experiments [79,80,81,82]. A network linking genes by their co-expression was generated [83] where each gene is represented by a node and each instance of high correlation among two nodes by an edge. Within these networks, the position of a gene within the total reflects its centrality, measured in degrees and betweenness. The degree refers to the number of edges a node has with other nodes, and the betweenness is a measure of how much a single node connects two larger clusters of nodes. The network inferred for *N. gonorrhoeae* contained ~1000 genes and included the six candidates (Figure S1A); NGO0416, NGO1043, NGO1215 and NGO1701 had average centrality values for both degree and betweenness, NGO690 had a high betweenness value and NGO0948 had both high degree and high betweenness values (Figure S1B). Information on a gene’s centrality adds to its potential as putative vaccine target, but lower or even lack of centrality does not warrant exclusion [84,85,86]. 

### 3.4. Candidate Antigens Cloning, Expression and Purification 

**Figure 2:**
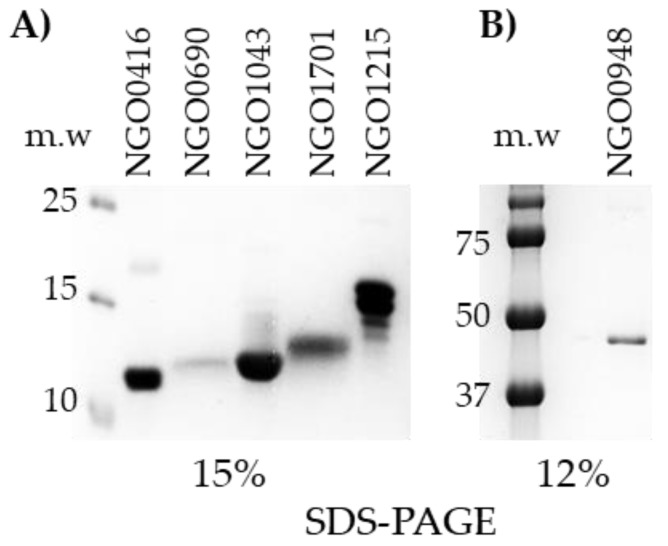
SDS-PAGE and Coomassie staining of purified candidates. (**A**) 15% SDS-PAGE. NGO0416, predicted molecular weight (m.w.) 10.8 kDa; NGO0690, predicted m.w. 12.1 kDa; NGO1043, predicted m.w. 10.2 kDa; NGO1701, predicted m.w. 14.4 kDa; NGO1215, predicted m.w. 16.1 kDa. (**B**) NGO0948, predicted m.w. 44.8 kDa.

NGO0416, NGO0690, NGO0948, NGO1043, NGO1215, NGO1701 were cloned as truncated proteins without the SP, and NGO0948 was cloned as a full-length protein. NGO1043, NGO1215 and NGO1701 were purified by Ni^++^ affinity chromatography in native condition (>50% soluble); NGO0416, NGO0690 and NGO0948 were purified in denaturing conditions from inclusion bodies. The positive fractions were identified by dot-blot analysis with a mouse anti-His antibody, pooled and dialyzed against PBS. The purified proteins were examined by SDS-PAGE and Coomassie staining on 15% (Figure 2A) or 12% acrylamide (Figure 2B) gels based on the predicted molecular weight of each protein.

### 3.5. Characterization of the Mouse Immune Responses to the Candidate Antigens 

#### 3.5.1. Total Antibody Responses 

**Figure 3:**
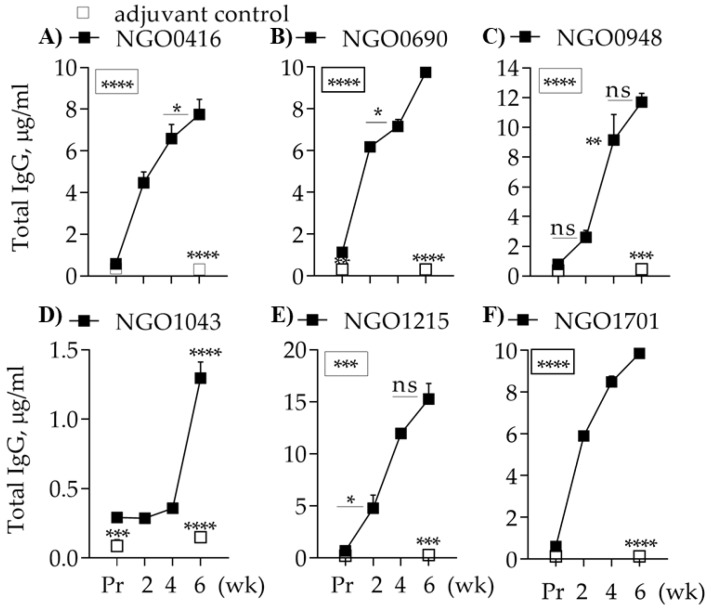
Total antibody production. Total IgG (μg/mL ± SEM) measured by ELISA of mouse preimmune (Pr) and immune sera (wk 2, wk 4 and wk 6) from individual mice (*n* = 8) tested in duplicate. (**A**) NGO0416; (**B**) NGO0690; (**C**) NGO0948; (**D**) NGO1043; (**E**) NGO1215 and (**F**) NGO1701 (black squares). *, **, ***, *** *p* significant (0.05 to < 0.0001) by one-way ANOVA with Tukey’s multiple comparisons test (boxed asterisks = significance for all time points). Pooled Preimmune and immune sera (wk 6) from the adjuvant control mice group (open squares) tested in quadruplicates. ***, **** *p* significant (0.005 to < 0.0001) by student t test vs. antigen-specific sera at same timepoints.

To verify the predicted immunogenicity of the candidates, the purified proteins were used to immunize female BALB/c mice sub-cutaneously with alum as an adjuvant. As a control, a separate group of mice was immunized with PBS/alum (adjuvant control). Pre-immune sera (Pr) and immune sera collected two weeks after each immunization (wk 2, wk 4 and wk 6) were tested for presence and amounts of antigen-specific antibodies by ELISA. NGO0416, NGO0690, NGO0948, NGO1215 and NGO1701 induced robust IgG production (Figure 3A–C,E,F) and NGO1043 induced a substantially lower IgG amount (Figure 3D). The adjuvant-control mice group did not mount an antibody response to the antigens (Figure 3A–F, open squares). 

#### 3.5.2. Antibody Subclasses

**Figure 4:**
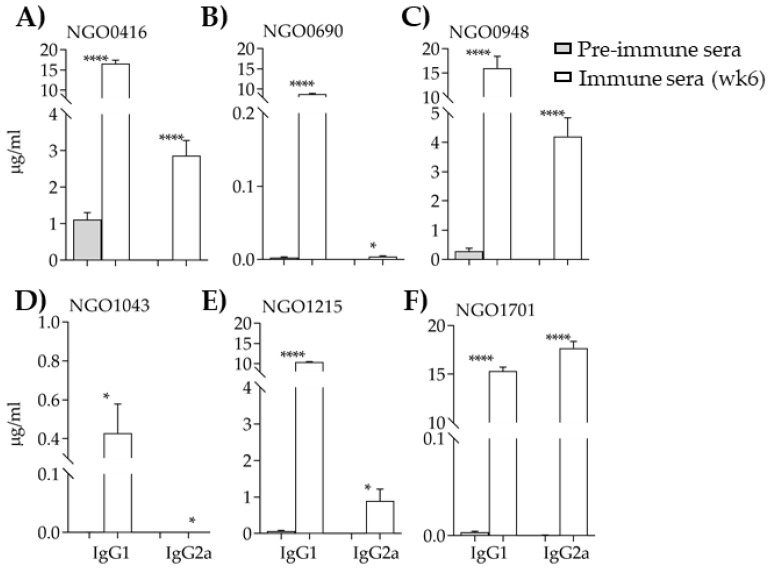
IgG subclasses. IgG1 and IgG2a (μg/mL ± SEM) measured by ELISA in preimmune (gray bars) and immune sera (wk 6) (white bars). (**A**) NGO0416; (**B**) NGO0690; (**C**) NGO0948; (**D**) NGO1043; (**E**) NGO1215 and (**F**) NGO1701. *, **** *p* significant (0.05 to < 0.0001) by student t test between preimmune and immune sera.

Analysis and quantification of the IgG subclasses in all sera revealed an abundant IgG1 profile, typically induced when using alum as an adjuvant (a Th2-type adjuvant) (Figure 4). Overall low antibody levels were measured in the anti-NGO1043 sera (Figure 4D). In the anti-NGO0416, anti-NGO0948 and anti-NGO1701 sera, production of IgG2b antibodies were also detected (Figure 4A,C,F). An IgG1/IgG2 ratio >1 was calculated for all the antigens except NGO1701 (IgG1/IgG2a = 0.86).

#### 3.5.3. Serum Cytokines

**Figure 5:**
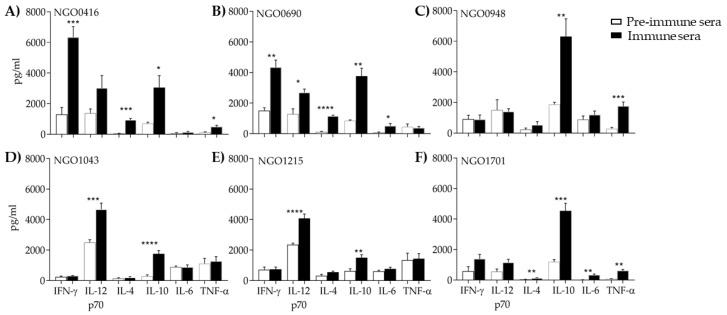
Serum cytokine profiles. Cytokine levels were measured by ELISA and quantified in pg/mL in preimmune sera (white bars) and immune sera (wk 6, black bars). (**A**) NGO0416, (**B**) NGO0690, (**C**) NGO0948, (**D**) NGO1043, (**E**) NGO1215 and (**F**) NGO1701. *, **, ***, **** *p* significant (0.05 to <0.0001) by student t-test vs. pre-immune sera.

The serum cytokine profile in the pre-immune and immune sera (wk 6) was examined by ELISA. In all immunized groups, IL-10 was significantly increased as compared to the pre-immune sera (Figure 5A–F, white bars and black bars, respectively). Moderate to high IL-12p70 levels were in the NGO0416, NGO0690, NGO1043 and NGO1215 groups (Figure 5A–E), with a slightly high background in the pre-immune sera (except for NGO1701, Figure 5F). However, normalization of the IL-12p70 levels in the immune sera to the pre-immune sera revealed comparable ratios for NGO0146, NGO0690, NGO1043 and NGO1215 (ratio between 2.1 and 1.72) to that of NGO1701 (ratio of 2), where the pre-immune was not elevated. Based on the antagonistic and interdependent function of IL-10 and IL-12 [87], higher IL-10 and concurrently lower IL-12p70 observed for the NGO0948 and NGO1701 groups were not surprising (Figure 5C,F, respectively). Elevated IFN-γ levels compared to unimmunized mice were only measured in the NGO0416 and NGO0690 sera groups (Figure 5A,B). Generally low levels of IL-6 and TNF-α were observed, although statistical significance was measured for TNF-α production in the NGO0416, NGO0948 and NGO1701 groups as compared to pre-immune sera (Figure 5A–F). Since all the antigens were used with the same adjuvant, the observed variability in the cytokine levels and profile may be due to a potential antigen-dependent effect.

#### 3.5.4. Antibody Cross-Reactivity with *N. gonorrhoeae* Strains

**Figure 6:**
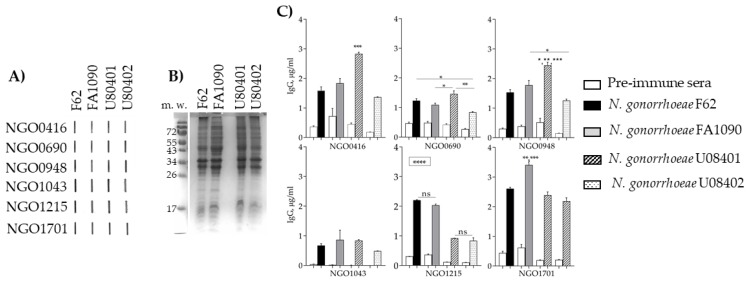
Mouse sera cross-reactivity with whole *N. gonorrhoeae* organisms. (**A**) Immunoblot of FF *N. gonorrhoeae* strains F62, FA1090, U80401 and U80402 (2–4 × 10^8^ total organisms/slot) with pooled immune sera (wk 6) to each candidate (1:200 dilution). (**B**) SDS-PAGE and Coomassie staining of bacteria as in (**A**). (**C**) Whole bacteria ELISA. Each panel shows the total IgG (μg/mL ± SEM) measured in each mouse antisera (triplicate pooled sera aliquots, week 6, 1:100) against FF *N. gonorrhoeae* strains F62 (black bars), FA1090 (gray bars), U08401 (striped bars) and U08402 (dotted bars). Preimmune sera (white bars). *, **, ***, **** *p* significant (0.05 to <0.0001) by one-way ANOVA with Tukey’s multiple comparisons test (boxed asterisks = significance for all samples).

To verify immune recognition of whole bacteria by the mouse antisera, formalin fixed (FF) *N. gonorrhoeae* strains F62 and FA1090 and two strains from our Nanjing cohort collection, U08401 and U08402 [43,44] were used. First, a qualitative analysis was carried out by immunoblotting, revealing that the four strains were recognized by the antisera to all the candidates (Figure 6A). This crude analysis showed some minor variability in the immunoblot intensity among the strains, possibly due to slightly different expression levels of each candidate in the different organisms. The equivalent bacteria amount from the same O.D. cultures were examined by SDS-PAGE and Coomassie staining to verify the total protein content spotted for the immunoblot, showing consistency among the major protein bands (Figure 6B). For a more quantitative analysis of immune recognition and cross-reactivity, a whole-cell ELISA was used. Very similar levels of anti-NGO0690 and anti-NGO1043 cross-reactive IgGs (µg/mL ± SEM) were measured among the four strains (Figure 6C); the anti-NGO0416 and anti-NGO0948 sera recognized *N. gonorrhoeae* strain U08401 significantly better than the other strains (Figure 6C, striped bars), the anti-NGO1215 sera recognized *N. gonorrhoeae* strains F62 and FA1090 significantly better than the clinical strains (Figure 6C, black and gray bars), and the anti-NGO1701 antibodies were significantly higher against *N. gonorrhoeae* strain FA1090 (Figure 6C). These results confirmed expression and immune recognition of the six candidates in different *N. gonorrhoeae* strains and indicated that expression may vary among strains.

### 3.6. Sub-Cellular Localization of the Candidate Antigens 

**Figure 7:**
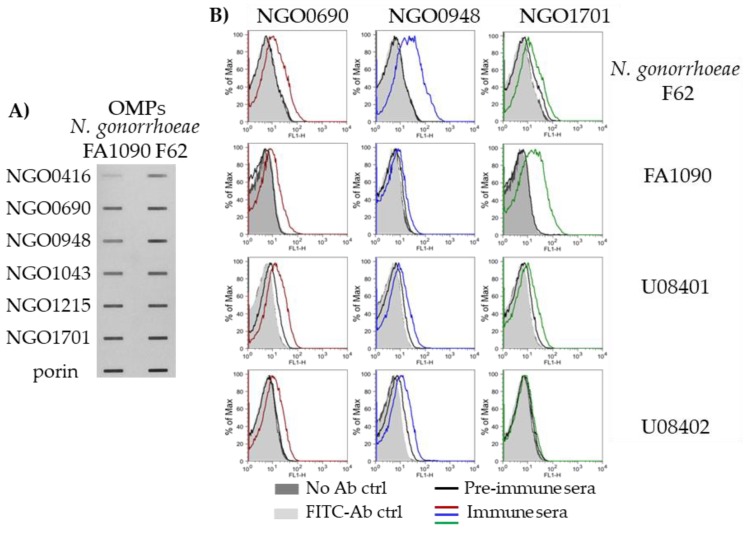
Sub-cellular localization and membrane surface expression in whole *N. gonorrhoeae* organisms. (**A**) Immunoblot of OMPs from *N. gonorrhoeae* strains F62 and FA1090 (5 µg/slot) with pooled immune sera (wk 6) to each candidate (1:200 dilution) and an anti-porin antibody control. (**B**) Bacterial surface localization of the candidates measured by flow cytometry of formalin fixed (FF) *N. gonorrhoeae* strains F62, FA1090, U80401 and U80402. Anti-NGO0690 mouse pooled sera (red line histograms), anti-NGO0948 (blue line histograms) and anti-NGO1701 (green line histograms); pre-immune sera (black line histograms), FITC-labeled secondary anti-mouse IgG control antibody (light gray histograms) and unstained bacteria controls (dark gray histograms). Each histogram is representative of triplicate experiments.

To verify the predicted cellular localization of the six candidates, outer membrane protein (OMP) fractions were prepared from *N. gonorrhoeae* F62 and FA1090 and examined by immunoblot analysis as above. An anti-porin antibody was used as a representative OMP control (porins represent >60% of the neisserial OMPs). All antisera recognized purified OMPs from both *N. gonorrhoeae* strains (Figure 7A). The immunoreactivity of NGO0416 was more modest than that of the other candidates in both strains, and that of NGO0948 appeared lower in *N. gonorrhoeae* FA1090 than in *N. gonorrhoeae* F62. To verify recognition of the six candidates on the surface of free, intact bacteria, the antisera were tested by flow cytometry. FF *N. gonorrhoeae* strains F62, FA1090, U08401 and U08402 were incubated with each sera followed by a FITC-labeled secondary anti-mouse IgG antibody; pre-immune sera were used as a negative control (Figure 7B, thin line histograms), bacteria incubated only with the FITC-labeled secondary antibody (Figure 7B, light gray histograms) and without antibodies (Figure 7B, dark gray histograms) were baseline controls. No shift in fluorescence indicative of antibody binding to the bacteria surface was observed with the anti-NGO0416, anti-NGO1043 and anti-NGO1215 sera for any of the *N. gonorrhoeae* strains (not shown). In contrast, an increased fluorescence over the baseline and the negative controls was observed for the anti-NGO0690, anti-NGO0948 and anti-NGO1701 sera (Figure 7B). The anti-NGO0690 sera recognized the four strains similarly (Figure 7B, red line histograms), the anti-NGO0948 sera recognized *N. gonorrhoeae* F62 better than the other strains (Figure 7B, blue line histograms), and the anti-NGO1701 sera recognized *N. gonorrhoeae* strain FA1090 better (Figure 7B, green line histograms). These results confirmed sera cross-reactivity with different *N. gonorrhoeae* strains and that NGO0690, NGO0948 and NGO1701 were accessible to antibodies on the bacterial surface, although all candidates were at least in part, associated with the outer membrane. 

### 3.7. Antibody Bactericidal Activity 

To evaluate the ability of the antisera to kill *N. gonorrhoeae*, the serum bactericidal assay (SBA) was used, with IgG/IgM-depleted normal human serum (NHS) as a source of complement. Bacterial survival (CFUs T30/CFUs T0) was measured, killing was expressed as the inverse of survival (%) and killing titers as the reciprocal of the sera dilution that kills 50% or more bacteria. Incubation of bacteria with NHS alone (10% with *N. gonorrhoeae* F62, U08401 and U08402, 20% with *N. gonorrhoeae* FA1090) induced none to minimal killing (Table 3). IgM-depleted heat-inactivated sera from mice immunized with PBS/alum (adjuvant control) induced minimal killing of *N. gonorrhoeae* U08401 and U08402 strains (up to 15% at a 1/10 dilution) (Table 3), and anti-NGO0416 sera induced low killing of *N. gonorrhoeae* U08401 and FA1090 strains. The anti-NGO1043 sera induced a strain-specific and low-titered killing of *N. gonorrhoeae* strains F62, U08401 and U08402, but failed to kill strain FA1090; anti-NGO1215 sera only induced low-titered killing of *N. gonorrhoeae* strain U08401. The anti-NGO0948 sera consistently killed all strains, although the titers were still low titers (Table 3); lastly, the anti-NGO0690 and anti-NGO1701 sera also induced killing of all strains and had higher titers, particularly against *N. gonorrhoeae* strains F62 and U08402 (Table 3). 

**Figure 8:**
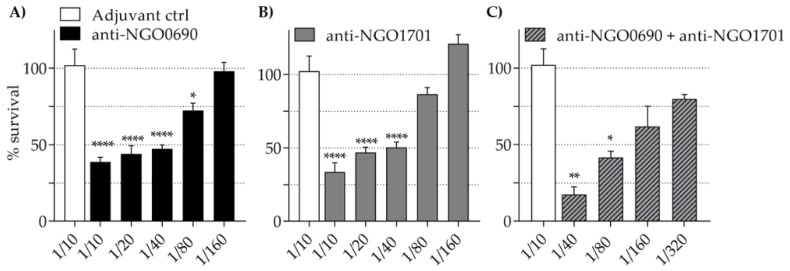
Serum bactericidal activity (SBA). Percent survival (CFU at T30/T0 ± SEM) of *N. gonorrhoeae* F62 incubated with serial dilutions of (**A**) anti-NGO0690 sera (black bars), (**B**) anti-NGO1710 sera (gray bars) and (**C**) anti-NGO0690 + anti-NGO1701 sera (gray striped bars) in the presence of 10% normal human serum (NHS). (A–C) adjuvant control sera, white bars. *, **, **** *p* significant (0.05 to <0.0001) by one-way ANOVA with Dunnett’s multiple comparison test vs. the adjuvant control (white bars).

These two antisera were further investigated to determine whether the killing titers could be enhanced by sera combination. Co-incubation of anti-NGO0690 and anti-NGO1701 sera increased killing titers of *N. gonorrhoeae* strain F62 to 1/80–1/160 (Figure 8C, gray dashed bars) as compared to the individual sera (titer of 1/40 for the individual sera) (Figure 8A,B, black and gray bars, respectively), suggesting an additive effect.

## 4. Discussion

We have designed a customized high throughput in silico pipeline for gonococcal antigen discovery built on a reverse vaccinology approach integrated with bioinformatic tools, CASS (candidate antigen selection strategy), divided into discovery and analysis phases (DAP). The approach foundation was grounded in our previous observation that gonococcal genes are differently expressed during human natural mucosal infection than growth in vitro [43]. Environment-dictated gene regulation is well known in *Neisseriae*, for example for capsule, pili, Opas, iron-regulated proteins [80,88,89] and antimicrobial resistance genes during natural human infections [43,44]. While CASS is applicable to any class or group of proteins, we have focused on proteins classified as hypothetical in our transcriptome study [43] as a group of untapped potential novel antigens not yet examined because they have likely evaded detection in vitro (most factors contributing to gonococcal disease have been identified in experimental conditions loosely representing the bacteria in their specific host environment). We mined these through serial cut-off criteria. 

The primary attribute was mRNA expression levels during natural human mucosal infection of both male and female subjects. mRNA levels and actual protein levels are subject to different regulatory processes but in bacteria there is correspondence between transcript levels and protein levels due to the direct coupling of transcription and translation [90]. Genes expressed at <50 RPKM were assigned lower priority because, even if they may induce protective immune responses when used in vaccinations, a low protein expression in vivo may dampen the efficacy of such response and also complicate analyses in in vitro-based platforms (i.e., ELISA, FACS, SBA). However, we do not negate their potential as antigens. There are other limitations to the approach, for example proteins down-regulated during infection may be critical for bacteria immune evasion mechanisms, proteins that are highly expressed may induce non-protective antibodies (see Rmp) or may be only important for metabolism and bacterial growth. The mRNA level cut-off also excluded gonococcal proteins that were expressed at different levels during infection of males and females (i.e., RPMK was <50 in one and >50 in the other). However, the pool of potential universal vaccine candidates is enriched. Subsequent CASS attributes converged on crucial antigen requirements by applying predictive tools for protein immunogenicity and membrane localization. The latter are not 100% accurate, but most cytosolic or secreted proteins can be sorted by combining localization, topology and function predictions. An exclusion attribute was also amino acid sequence conservation in common commensal species (to avoid wide-spread immunity to unrelated bacteria), in humans and in mice (to avoid potential self-reactive antigens that may induce auto-immunity and/or interfere with pre-clinical vaccine studies). Protein sequence conservation among strains within the same bacterial species, however, is critical for broad protection. In *Neisseria*, sequence conservation is a double-edged sword: proteins that are very conserved or highly variable have both failed as vaccine candidates because they may not induce robust adaptive or protective immune responses (i.e., Rmp), or not protect against heterologous strain infections (i.e., pilin). A >40% amino acid sequence conservation over >70% of the total protein sequence length in the *N. gonorrhoeae* protein sequences database was considered as an indication of potential cross-reactivity in a broad range of gonococcal strains. “Good” antigens should also have structure favorable for recombinant production, and attributes desirable for manufacturability were prioritized to avoid future limitation to only pre-clinical analyses (an example is the *Chlamydia* outer membrane protein MOMP, an excellent protective antigen in *Chlamydia* experimental models of infection, but unsuitable for recombinant production due to refolding issues [91]). Proteins with high structure hierarchy (i.e., number of trans-membrane domains) were given low priority, enriching the group of antigens with low to moderate structure constrains; ultimately, those that are surface-exposed would be likely accessible to antibody recognition for complement-dependent bacterial killing, opsonophagocytosis or inhibition of bacterial adhesion to host cell and colonization. However, periplasmic proteins may be partially surface-exposed and are present in (either naturally released by bacteria or induced) outer membrane vesicles (OMV). The meningococcal OMV-based vaccine Bexsero contains both OMPs and periplasmic proteins [92,93,94]; this vaccine has been recently shown to be partially protective against *N. gonorrhoeae* in vaccinated subjects and in animal models, likely attributable to conserved and cross-reactive antigens [36,37,38,39]. For the same reason, proteins predicted to be associated with the inner membrane but facing the periplasm could also be potential candidates. 

Thirty-six targets were identified by CASS: 21 proteins with potential functions and 15 with unknown function; we began immunological characterization of six purified recombinant candidates. Three were predicted lipoproteins, NGO0690 (hypothetical), NGO0948 (NlpB, BamC homolog) and NGO1043 (hypothetical, Ag473 homolog). Lipoproteins play a role in bacterial membrane metabolism and functions, in pathogenesis by mediating host cell adhesion and inflammatory responses and are considered good vaccine candidates [95]. Examples in *Neisseriae* include established vaccine targets (factor H binding protein (fHbp) part of both Bexsero and Trumenba [92,96]) and preclinical targets (reviewed in Reference [97]), for example TbpB [25], NMB0928 [70,73] (homolog of NGO0948), MetQ [30,34]. Two candidates were potentially involved in metal binding, NGO1215 (AccA [86]), and NGO1701 (hypothetical). Metal transport membrane proteins are important for bacterial metabolism, growth and pathogenesis by providing host nutritional immunity escape strategies [98]. Iron requirements and the proteins associated with iron transport and metabolism in *Neisseriae* are well studied [99,100]; zinc, copper and other metals are also critical [101] along with proteins that modulate their functions, such as ZnuD, TdTs and CopA [102,103,104,105] to mention a few. NGO0416 did not have major evidence of function. These candidates were part of the *N. gonorrhoeae* core genome [62] and had a relatively small range of allelic variability. In addition, they lacked allelic overlap in *N. lactamica* (the *ngo0416* gene was absent in *N. lactamica*) and showed limited protein sequence identity within the reservoir of *N. lactamica* protein databases. *N. lactamica* colonizes the nasopharynx at an early age and likely contributes to natural immunity to *N. meningitidis* and meningococcal carriage [106]; in case of interference by anti-gonococcal immunity, these natural immune responses may be attenuated. Although robust responses to this commensal organism should be unlikely, further analyses will be necessary to verify lack of cross-reactivity. Analysis of predicted centrality in a *N. gonorrhoeae* gene co-expression network (McClure et al., submitted) provided additional rationale for the candidates, especially NGO0690 and NGO0948. Centrality has been used as a metric to rank gene importance in a biological system from bacterial growth and metabolism, infection, to even cancer [84,85,86,107]. Information on a gene centrality and edges can also assist in annotation/assignment to a defined module based on function enrichment, a process termed Guilt-by-Association (GBA). This sophisticated approach has been used to predict functions of unknown genes based on the edges they have with well-characterized genes in the network [108] and may offer context for genetic manipulation and characterization of the targets in future studies.

Despite the challenges associated with recombinant expression and folding of proteins with limited or no structure information, most of the purified antigens elicited robust antibody production in mice with a generally Th2-type bias (high IgG1 levels and mostly IL-10) and low inflammatory responses. Induction of IgG2a, or IL-12p70 and IFN-γ by some candidates despite the immunizations were carried out with alum, suggested intrinsically different properties of the candidates. For example, some candidate may induce activation of PRR (pattern recognition receptors)-mediated signaling pathways. Since Th1 responses contribute to clearance of infection and lead to protective memory response in a gonococcal mouse infection model [109,110,111], presence of a Th1 component may be advantageous for a tailored and robust anti-gonococcal immunity and could be exploited using different adjuvants (possibly mucosal). Sera cross-reactivity with different gonococcal strains suggested presence of heterologous cross-reactive epitopes. Differences in the magnitude of immune recognition of these organisms may suggest that expression levels of each candidate naturally vary among strains, but it is also possible that these candidates are down-regulated in vitro. The degree of surface exposure and accessibility to antibodies on the bacterial surface may also be different in substrate-bound bacteria and free bacteria. Any of these hypotheses may explain the lack of concordance of the ELISA/blot analyses and the FACS analysis for some of the candidates. The discrepancy between the OMP immunoblot and FACS could also be an artifact of the OMP extraction procedure. The Sarkosyl method is reported to solubilize inner-membrane proteins [112]; it is possible that predicted periplasmic proteins with a loose association with the IM may be retained, but are not actually surface-accessible in free whole bacteria. These may not represent desirable candidates, especially if they fail to induce protective responses. The correlates of protection for gonorrhea are not known, but assays such as complement-dependent antibody-mediated bactericidal killing, opsonophagocytic killing or inhibition of host cell adhesion/invasion at the colonization site are most used in vitro. In the serum bactericidal assay (SBA), antibodies to NGO0416 had negligible bactericidal activity and those to NGO1215 only killed one *N. gonorrhoeae* strain, reflecting scarce potential as a broad vaccine target. Despite not being recognized as surface-exposed in free bacteria, anti-NGO1043 antibodies had low-titered bactericidal activity against three diverse *N. gonorrhoeae* strains, possibly due to any of the reasons discussed above. In contrast, sera raised to NGO0948, NGO0690 and NGO1701 were bactericidal against all the strains tested; despite the killing titers were not very high, these results are promising. Indeed, anti-NGO0690 and anti-NGO1701 sera combination led to an increased killing titer, suggesting that immunization with multiple antigens may induce a more potent protective immune response. Analyses of protective responses in a mouse model of gonococcal infection and construction of deletion mutants is ongoing and will clarify the role of these candidates in anti-gonococcal immunity and in *N. gonorrhoeae* metabolism and pathogenesis.

In conclusion, inclusion of information on gonococcal protein expression during natural infections in humans, when available, will provide new insights for more sophisticated and targeted strategies for identification and selection of vaccine candidates. 

## 5. Patents 

Massari, Paola et al. Title: VACCINE COMPOSITIONS AND METHODS OF SELECTING ANTIGENS. U.S. Application No.: 62/881,627. Filed: 1 August 2019. 

## Figures and Tables

**Table 1:** CASS summary of the six gonococcal candidate antigens.

Candidate	mRNA Expression In Vivo (RPKM)		PREDICTED								**Core Genome ^5^**
			Antigen Score ^1^	Localization ^2^	Structure ^3^				Function ^4^		
					TM Domain	Signal Sequence		Size (kDa)	Annotation/Homology		
NGO0416	196	127	0.5	P, non-cytosol	0	SP	10.8		hypothetical	NEIS0782NMB0844	yes
NGO0690	142	157	0.7	P/OM, non-cytosol	1	LP	12.1		hypothetical, lipoprotein	NEIS1164NMB1047	yes
NGO0948	125	105	0.43	P/OM, non-cytosol	0	-	44.8		lipoprotein, NlpB/DapX/BamC	NEIS0906NMB0928	yes
NGO1043	1748	519	0.82	P/OM, non-cytosol	0	LP	10.2		hypothetical, lipoprotein	NEIS2446NMB1468	yes
NGO1215	190	292	0.67	P, non-cytosol	0	SP	16.1		hypothetical, PCu(A)C/, AccA	NEIS1474NMB1557	yes
NGO1701	127	121	0.41	P, non-cytosol	0	SP	14.4		membrane, TAT_Cys_rich four helix bundle copper-binding protein	NEIS2720	yes

^1^ VaxiJen; ^2^ PSORTb, PredictProt, Gneg-mPLoc, Phobius; ^3^ TMHMM, SignalP, LipoP; ^4^ BLASTp, UniProtKB, PFAM, KEGG, PubMLST, Vaxign; ^5^ PubMLST.

**Table 2:** Candidates alleles analysis in *N. gonorrhoeae* (4198 strains in PubMLST).

Gene	Total Alleles	Frequency ^1^		Distribution ^2^ (% of 4198 Strains)	*p*-Distance	Polymorphic Sites (Total)	Polymorphic Sites ^3^	
		Allele	# of Strains				Allele	Non-Synonimous Mutations
*ngo0416 (NEIS0782)*	6	17	3069	73.7	0.006	5	-	-
		26	124	2.9			-	-
*ngo0690 (NEIS1164)*	17	7	3332	79.3	0.01	27	-	-
		32	815	19.4			32	S37R
*ngo0948 (NEIS0906)*	55	12	2354	57	0.005	109	-	-
		28	363	8.6			28	A319R
		32	324	7.7			32	I18M, T19K
		214	248	5.9			214	Q49R
*ngo1043 (NEIS2446)*	27	57	2720	64.7	0.01	26	-	-
		64	691	16.4			64	V65A
		58	283	6.7			58	V65A, A116T
		80	262	6.2			80	V65A
*ngo1215 (NEIS1474)*	13	13	3817	91	0.012	27	13	x143P
		16	273	7			16	x143S
*ngo1701 (NEIS2720)*	11	1	3379	80.4	0.005	10	-	-
		2	803	19.1			2	E126A

^1^ Most frequent alleles in the highest number of strains; ^2^ Percent of strains expressing the high frequency alleles^1^ within the total number of isolates. Data collected from PubMLST [61]; ^3^ Polymorphisms in the most frequent alleles^1^.

**Table 3:** Serum bactericidal assay. Bacterial killing ^a^ and serum titers ^b^.

	*N. gonorrhoeae* Strains			
Sera Groups	F62	U08401	U08402	FA1090 ^c^
NHS alone	3%	6%	3%	0%
Adjuvant control	0% (1/10)	14% (1/10)	8% (1/10)	0% (1/10)
NGO0416	9% (1/10)	28% (1/10)	8% (1/10)	30% (1/5)
NGO0690	53% (1/40)	50% (1/10)	46% (1/20)	45% (1/10)
NGO0948	50% (1/10)	50% (1/10)	52% (1/10)	57% (1/5)
NGO1043	46% (1/10)	55% (1/10)	48% (1/10)	13% (1/5)
NGO1215	17% (1/10)	57% (1/10)	23% (1/10)	0% (1/5)
NGO1701	50% (1/40)	55% (1/10)	46% (1/20)	52% (1/5)

^a^ % inverse of survival (CFU T0/CFU T30); ^b^ reciprocal of the sera dilution that kills 50% or more bacteria; ^c^ 20% NHS.

## Supplementary Materials

The following are available online at https://www.mdpi.com/2076-393X/7/4/153/s1, Figure S1: Network analysis. Table S1: Candidates alleles analysis in *N. lactamica* (288 strains in PubMLST).
