# Supplementary material for: Integrated Bioinformatic Analyses and Immune Characterization of New Neisseria gonorrhoeae Vaccine Antigens Expressed during Natural Mucosal Infection
Source: Vaccines (Basel). 2019 Oct 17;7(4):153. doi: 10.3390/vaccines7040153 (PMC6963464; doi:10.3390/vaccines7040153)
Supplement: Supplementary file 1 [file vaccines-07-00153-s001.pdf]

Supplementary Materials:

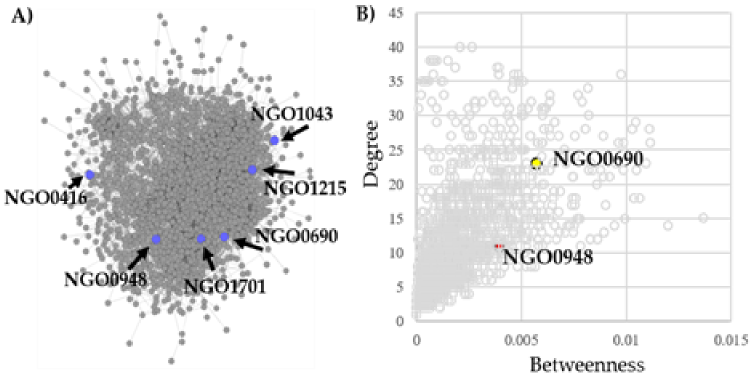

Figure S1. Network analysis. (**A**) The six candidates positioned within the gonococcal gene expression network. (**B**) Centrality of the candidates within the gonococcal gene network measured in degree (y-axis) and betweenness (x-axis) values. NGO0690 (yellow dot) has both high degree and betweenness and NGO0948 (red dot) has high degree. The other four candidates have average values (not shown).

Table S1. Candidates alleles analysis in *N. lactamica* (288 strains in PubMLST).

Gene	Total Alleles	[1]Frequency		[2]Distribution (% of 288 strains)	*p*-Dist.
		Allele	# of Strains		
ngo0416 (*NEIS0782*)	-	-	288	0	0.006
ngo0690 (*NEIS1164*)	13	3<br>8	229<br>23	79.5<br>7.9	0.01
ngo0948 (*NEIS0906*)	17	3<br>70	225<br>21	78.1<br>7.2	0.005
ngo1043 (*NEIS2446*)	8	28<br>17	242<br>21	84<br>7.3	0.01
ngo1215 (*NEIS1474*)	10	34<br>7	241<br>23	74.3<br>7.9	0.012
ngo1701 (*NEIS2720*)	10	20<br>24	225<br>23	78.1<br>7.9	0.005

[1] Most frequent alleles in the highest number of strains. [2] Percent of strains expressing the high frequency alleles[1] within the total number of isolates. Data collected from PubMLST [61].
